# Development of EndoScreen Chip, a Microfluidic Pre-Endoscopy Triage Test for Esophageal Adenocarcinoma

**DOI:** 10.3390/cancers13122865

**Published:** 2021-06-08

**Authors:** Julie A. Webster, Alain Wuethrich, Karthik B. Shanmugasundaram, Renee S. Richards, Wioleta M. Zelek, Alok K. Shah, Louisa G. Gordon, Bradley J. Kendall, Gunter Hartel, B. Paul Morgan, Matt Trau, Michelle M. Hill

**Affiliations:** 1 QIMR Berghofer Medical Research Institute, Herston, QLD 4006, Australia; ja.webster@connect.qut.edu.au (J.A.W.); Renee.Richards@qimrberghofer.edu.au (R.S.R.); alok.shah@csl.com.au (A.K.S.); Louisa.Gordon@qimrberghofer.edu.au (L.G.G.); bradley.kendall@uq.edu.au (B.J.K.); Gunter.Hartel@qimrberghofer.edu.au (G.H.); 2 Centre for Personalised Nanomedicine, Australian Institute for Bioengineering and Nanotechnology (AIBN), The University of Queensland, Brisbane City, QLD 4072, Australia; a.wuethrich@uq.edu.au (A.W.); k.shanmugasundaram@uq.edu.au (K.B.S.); m.trau@uq.edu.au (M.T.); 3 Division of Infection and Immunity, Cardiff University, Heath Park, Cardiff CF10 3AX, UK; ZelekW@cardiff.ac.uk (W.M.Z.); morganbp@cardiff.ac.uk (B.P.M.); 4 Faculty of Medicine, The University of Queensland, Herston, Brisbane, QLD 4102, Australia; 5 Department of Gastroenterolgy and Hepatology, Princess Alexandra Hospital, Brisbane, QLD 4102, Australia; 6 School of Chemistry and Molecular Biosciences, The University of Queensland, St Lucia, QLD 4072, Australia

**Keywords:** Barrett’s esophagus, biomarker, surveillance, screening, surface-enhanced Raman spectroscopy, SERS, complement component, liquid biopsy, lectin, glycoprotein

## Abstract

**Simple Summary:**

Esophageal adenocarcinoma (EAC) is often detected late and has a poor survival rate. Currently, patients are selected for endoscopy-biopsy diagnosis based on clinical risk factors such as the precursor condition Barrett’s esophagus, history heartburn/reflux, age and high body mass index. To enable blood-based screening, we previously discovered and validated novel blood biomarkers for early stage EAC. To support clinical application, here, we report the technology development of the microfluidic EndoScreen chip and validation using the biomarker JAC-C9 (Jacalin-lectin binding complement component C9) in a test cohort of 46 samples. Compared to clinical risk factors alone, we found that use of blood biomarkers JAC-C9 and total C9 in addition to clinical risk factors improved EAC prediction in this cohort, suggesting that a simple blood test can help the physician prioritize patients for endoscopic evaluation. Future work will deploy a panel of markers on a point-of-care version of EndoScreen chip, to enable population screening and early diagnosis of EAC and thereby reduce mortality from this cancer.

**Abstract:**

The current endoscopy and biopsy diagnosis of esophageal adenocarcinoma (EAC) and its premalignant condition Barrett’s esophagus (BE) is not cost-effective. To enable EAC screening and patient triaging for endoscopy, we developed a microfluidic lectin immunoassay, the EndoScreen Chip, which allows sensitive multiplex serum biomarker measurements. Here, we report the proof-of-concept deployment for the EAC biomarker Jacalin lectin binding complement C9 (JAC-C9), which we previously discovered and validated by mass spectrometry. A monoclonal C9 antibody (m26 3C9) was generated and validated in microplate ELISA, and then deployed for JAC-C9 measurement on EndoScreen Chip. Cohort evaluation (*n* = 46) confirmed the expected elevation of serum JAC-C9 in EAC, along with elevated total serum C9 level. Next, we asked if the small panel of serum biomarkers improves detection of EAC in this cohort when used in conjunction with patient risk factors (age, body mass index and heartburn history). Using logistic regression modeling, we found that serum C9 and JAC-C9 significantly improved EAC prediction from AUROC of 0.838 to 0.931, with JAC-C9 strongly predictive of EAC (vs. BE OR = 4.6, 95% CI: 1.6–15.6, *p* = 0.014; vs. Healthy OR = 4.1, 95% CI: 1.2–13.7, *p* = 0.024). This proof-of-concept study confirms the microfluidic EndoScreen Chip technology and supports the potential utility of blood biomarkers in improving triaging for diagnostic endoscopy. Future work will expand the number of markers on EndoScreen Chip from our list of validated EAC biomarkers.

## 1. Introduction

Population screening programs for breast and cervical cancers have successfully reduced mortality by detecting these cancers at an earlier stage, motivating the development of similar programs for other cancers. An effective cancer screening program is predicated on the identification of a defined high-risk population and the availability of a sensitive, cost-effective, minimally invasive screening test. In this work, we aim to translate serum biomarkers for early stage esophageal adenocarcinoma (EAC) into a blood test to better stratify patients for the gold standard diagnosis by upper endoscopy-biopsy, an invasive, costly and time-consuming procedure unsuitable for population screening [1].

EAC is thought to develop as a consequence of chronic gastro-esophageal reflux disease (GERD), with the metaplastic condition Barrett’s esophagus (BE) being the only known precursor condition [2,3]. To detect EAC at an early stage, BE patients and those with multiple risk factors for BE and EAC, such as age > 50 years, male sex, GERD history, acid suppression medication and high body mass index (BMI), are recommended to undergo endoscopic screening [4,5,6,7,8]. Despite improved risk factor identification and a BE surveillance program, temporal epidemiological data show that there has been no change in the proportion of people diagnosed into each stage of EAC since the 1970s [4]. Patients with EAC face a poor prognosis, with 5-year survival of less than 20% [9,10]. Detection at an early stage significantly improves survival, exemplified by the 48% 5-year survival rate for localized EAC [10]. Hence, there is an urgent need to improve early EAC risk stratification and diagnosis.

BE is asymptomatic and under-diagnosed. A recent meta-analysis reported that only ~12% of EAC patients had a prior diagnosis of BE, while ~57% had BE diagnosed at the same time as EAC [11]. To improve BE detection, several novel cytology sampling methods and biomarkers are at late stages of clinical development, including CytoSponge, EsophaCap and EsoCheck [2,12]. For BE patients who have undergone endoscopy with biopsy, TissueCypher and BarreGEN predicts EAC risk using a 15-marker immunohistochemistry panel and gene mutational load, respectively [2]. While these tests will assist BE diagnosis and risk stratification in the future, for the ~30% of EAC patients without prior or concomitant BE, there remains a need for screening biomarkers that detect EAC in a high-risk population, including patients in BE surveillance programs.

Based on the role of glycosylation in carcinogenesis, and the reported glycan changes during BE progression to EAC [13], we embarked on a glycoproteomics program to discover serum glycoprotein biomarkers for early EAC. Our pipeline used a panel of naturally occurring glycan-binding proteins, lectins, as affinity agents for different glycoforms [14], and tandem mass spectrometry methods for protein identification and quantitation [15]. Through a phased biomarker program with four patient cohorts from Australia and the USA (*n* > 350), we discovered and validated a panel of serum glycoproteins that can detect EAC at an early stage [15,16].

To develop the EAC serum glycoprotein biomarker panel for routine screening and monitoring of patients, a diagnostic technology for dual detection of the glycan and the protein is required. Furthermore, it needs to have high sensitivity and specificity in a serum background, ability for multiplexing a biomarker panel and ideally require minimal sample input and sample preparation. In this context, surface-enhanced Raman scattering (SERS) integrated in microfluidic systems have shown promise for sensitive biomarker panel detection using specific antibodies [17]. SERS is based on Raman scattering of light in proximity to plasmonic structures such as metallic nanoparticles [18]. The plasmonic structures enhance the relatively weak Raman signal by multiple orders of magnitude, enabling ultrasensitive biomarker analysis [19]. We previously integrated SERS with functional gold nanoparticles to microfluidic systems with asymmetric electrodes for detecting various biomarkers in liquid biopsies [20]. The small dimensions of the microfluidic systems allowed low sample input while providing a high surface area for sensing to further improve the detection sensitivity. Furthermore, under the effect of an alternating current field, the asymmetric electrodes stimulated a nanoscopic shearing on the sensor surface, which reduced non-specific binding of non-targets. Our previous work used single antibody for biomarker detection, but the technology could be adapted for dual detection of particular glycoforms of glycoproteins using a lectin and a protein-specific antibody.

As proof of concept for lectin immunoassay on the nanoshearing microfluidic SERS functionalized nanoparticle system, we chose the most robust EAC biomarker candidate, JAC lectin-binding complement component C9 (C9), with a view to multiplex detection of several glycoprotein biomarkers in the future. C9 is a glycoprotein of the terminal complement pathway, which functions to induce cytotoxicity by forming the membrane attack complex on target cell membranes. Our glycoproteomics studies confirmed elevated levels of four different circulating C9 glycoforms that bind to AAL, EPHA, JAC and NPL lectins, respectively [15,16]. In addition, we detected increased staining of C9 protein in BE and EAC tissues by immunohistochemistry [16], although we were not able to distinguish glycoforms on tissue.

To ensure supply of high quality, C9-specific antibodies for assay development and translation, we developed and validated a new C9 monoclonal antibody using standard enzyme linked immunoassay (ELISA). Using the new C9 monoclonal antibody, the JAC-C9 assay was established on the microfluidic platform with JAC lectin for capture and SERS-tagged C9 antibody for detection. The performance of JAC-C9 and total C9 for detection of BE and EAC was compared to gold standard endoscopy-biopsy diagnosis in a cohort of 46 participants. Finally, the diagnostic value of the small serum biomarker panel above baseline clinical parameters was evaluated in multimodal logistic regression models.

## 2. Materials and Methods

### 2.1. Study Cohort and Experimental Design

The study was approved by the Human Research Ethics Committee of QIMR Berghofer Medical Research Institute, Brisbane, Australia. A subset of the previously analyzed serum samples [15] were selected based on sample availability and were originally selected from the Study for Digestive Health [21,22]. All selected participants were male, in line with the male-predominance of BE and EAC [23]. Patient categorization into Healthy, BE or EAC was confirmed histologically, and age was balanced across the three groups (Table 1). Serum was taken at the time of endoscopic sample collection. Healthy controls had no history of esophageal cancer and no evidence of esophageal histological abnormality at the time of sample collection.

For total serum C9 ELISA, the samples were randomized across the ELISA plates. For analysis of JAC-C9 in serum, samples were analyzed blindly in conducting the assay. The method of modelling was determined prior to experimental analysis.

### 2.2. Recombinant Protein Expression and Purification

A human embryonic kidney 293 (Hek293) cell line stably expressing His-human C9 was generated using a human C9 pSectag2a plasmid kindly provided by Michelle Dunstone (Monash University, Melbourne, Australia) [24]. Hek293 cells were maintained in DMEM/Nutrient Mixture F-12 Ham (Sigma, North Ryde, Australia) in 2.5% heat inactivated fetal bovine serum (Gibco/Thermofisher Scientific, Seventeen Mile Rocks, Australia). C9-expressing Hek293 cells were seeded into HYPERFlask^®^ (Corning, NY, USA) and cultured until 90% confluent. Media was replaced with DMEM/F12 containing 5 mL of Hank’s buffered saline, 1× ITS (insulin–transferrin–sodium selenite) (Sigma) and 3 µM of sodium butyrate and cells were allowed to secrete protein for 3 days.

His-C9 was purified from collected culture media using an AKTA fast protein liquid chromatography (FPLC) with HisTrap excel 5 mL columns (Cytiva, North Ryde, Australia). Columns were equilibrated using 20 mM of sodium phosphate/0.5 M sodium chloride; washed using 20 mM of sodium phosphate/0.5 M sodium chloride/20 mM imidazole; and eluted using 20 mM of sodium phosphate/0.5 M sodium chloride/500 mM imidazole. Fractions containing protein were identified using a Bradford assay (Bio-Rad, Gladesville, Australia), pooled and dialyzed using 3.5 K MWCO snakeskin dialysis tubing (Thermo Fisher Scientific, Seventeen Mile Rocks, Australia) against 3 L of phosphate buffered saline (pH 7.4), replaced three times for 24 h. Protein expression was confirmed using Western blot and protein concentration was determined using SDS-PAGE with colloidal Coomassie using a bovine serum albumin (BSA) standard, and then verified again by nanodrop.

### 2.3. Antibody Production and Purification

Mouse monoclonal antibody (mAb) to human C9 was generated by immunization of wild type mice C57BL/6J, bred in-house) with human C9 using standard schedules [25]. Immunized mice were screened for antibody responses by ELISA and mice with the highest titre response were selected and re-boosted before sacrificing and harvesting of spleens. The splenocytes were fused with SP2 myeloma and aliquots were placed in 96-well plates. Positive hybridomas were selected by direct ELISA on immobilized human C9. The C9-positive mAb secreting clones were subcloned by limiting dilution to monoclonality. Three sub-clones of clone 26 were expanded: 2G6, 3C9 and 4G2, from which 3C9 was chosen for characterization and cultured in an Integra bioreactor (Sigma Aldrich, North Ryde, Australia, #Z688029-3EA) for large scale production of antibody.

The mAb was purified using 5 mL HiTrap Protein G sepharose columns (GE Healthcare, #GE17-0405-01). The column was washed and equilibrated with phosphate buffered saline (PBS), then IgG-containing Integra supernatant was applied at 1–2 mL/min, the column was washed with 50 mL of PBS and bound antibody eluted with 5 mL of 0.1 M glycine pH 2.5, collecting 1 mL fractions into 100 µL of 0.1 M Tri pH 7.2. Peak fractions were identified, pooled and dialysed overnight at 4 °C into PBS. Antibody concentration was measured using Bicinchoninic Acid (BCA) assay and the purified IgG was stored frozen in aliquots at >1 mg/mL. Mouse mAb was isotyped using IsoStrips (# 11493027001; Roche, North Ryde, Australia) as IgG1, *K*.

### 2.4. Serum Purified C9 and Production of Depleted Serum

C9-depleted serum and purified C9 standard were generated using affinity chromatography. Human serum was obtained from the Australian Red Cross Blood Service under QIMRB human ethics approval P2352. Serum from five healthy donors was pooled, diluted 1:1 with PBS then sterile filtered (0.22 µm). A 20 mL sample of the pooled serum was injected onto a 1 mL PBS-equilibrated HiTrap NHS-Activated HP affinity column (Cytiva, North Ryde, Australia) coupled to a monoclonal anti-C9 antibody [26]. C9-depleted serum fractions were collected in the flow-through and depletion confirmed by ELISA before pooling. The column was thoroughly washed with PBS before C9 was eluted with 5 mL of 0.1 M glycine, pH 2.5 and neutralized by addition of 0.5 mL of 1.5 M Tris, pH 8.8. Eluted C9 was desalted and buffer exchanged vs. PBS using a Zeba 7 K MWCO spin desalting column (Thermo Fisher Scientific) and concentrated using an Amicon ultra 10 K MWCO centrifugal filiter unit (Merck Millipore, Bayswater, Australia). C9 purity was assessed by SDS-PAGE and Western blot using a commercial anti-C9 antibody (ab17931, Abcam, Melbourne, Australia). Protein concentration of serum purified C9 was determined by BCA assay (Thermo Fisher Scientific).

### 2.5. Characterization of mAb 26 by Western Blot

Human C9 (in-house; 0.5 µg) was placed in wells and resolved on 4–20% sodium dodecyl sulphate–polyacrylamide gel electrophoresis gels (#4561093; Biorad, Hemel Hempstead, UK) under reducing^®^ and non-reducing (NR) conditions, then electrophoretically transferred onto 0.45 µm nitrocellulose membrane (Amersham/Cytiva, North Ryde, Australia). After transfer, membranes were blocked with 5% BSA in PBS-T, washed in PBS-T, cut into strips and incubated overnight at 4 °C with individual test sub-clone (2G6, 3C9, 4G2) each at 1 µg/mL in 5% BSA PBS-T. Bound test mAb was detected by incubation with donkey anti-mouse IgG-HRP (Jackson ImmunoResearch, West Grove, PA, USA, 715-035-150; 1:10,000 in 5% BSA PBS-T), developed with enhanced chemiluminescence (GE Healthcare) and visualized by autoradiography (Figure S1).

### 2.6. Hemolytic Assay

To test the function of purified C9, protein was added back to C9-depleted serum at various doses up to 70 µg/mL in undiluted C9-depleted serum (from the mAb 26 affinity column), then tested in hemolytic assay. Antibody-sensitized sheep erythrocytes (ShE; #ORLC25, Amboceptor Siemens, Dublin, Ireland) were suspended (2% *v/v*) in HEPES-buffered saline containing Ca^2+^ and Mg^2+^ (HBS^++^). Aliquots (50 µL) were placed into a 96-well round-bottomed plate followed by 50µL of the reconstituted C9-depleted serum diluted in HBS^++^, then 50 µL of HBS^++^ [27]. C9-depleted serum or NHS were used as controls. Plates were incubated at 37 °C for 30 min, centrifuged and hemoglobin in the supernatant measured by spectrophotometry (A405 nm). Percentage lysis was calculated according to: % Lysis = (A405 sample – A405 background)/(A405 max – A405 background) × 100%.

### 2.7. C9 Direct ELISA

MaxiSorp^TM^ ELISA plates (Sigma) were incubated overnight at 4 °C with serum or eluate from the C9 pull-down diluted in sodium carbonate buffer (pH 9). Known concentrations of purified recombinant His C9 were used as control standard. The following day plates were washed three times with PBS-T. The new monoclonal m26 3C9 anti-C9 antibody was biotinylated using Biotin (Type B; Fast Conjugation kit, Abcam) following manufacturer’s procedures. The plates were then incubated with 50 µL of biotinylated mouse anti-C9 antibody diluted in PBS-T/5% BSA (Sigma) at 2 µg/mL for 2 h at room temperature. Plates were then washed three times with PBS-T, followed by 30 min incubation at room temperature with 100 µL of Streptavidin-HRP (Abcam) diluted 1/2500. Plates were washed four times with PBS and developed using TMB (Thermo Fisher Scientific). When sufficient color was evident, development was stopped by adding 100 µL of 2 M phosphoric acid (Sigma Aldrich, North Ryde, Australia) and absorbance read at 450 nm. The standard was calculated by (absorbance − background) then plotted against the known concentration. Concentration was determined by inputting (absorbance − background) into the linear regression formula and multiplied by dilution factor.

### 2.8. EndoScreen Chip Fabrication and Functionalization

The microfluidic device was fabricated using standard photolithography and according to a previous report [28]. Briefly, the EndoScreen Chip with an asymmetric electrode array was fabricated on a borosilicate glass substrate. The array consisted of 28 electrodes arranged in 4 rows of 7 electrodes each. The array of the asymmetric circular ring electrodes was designed using layout L-Edit V15 (Tanner Research, Monrovia, CA, USA) and was written on 5-inch soda lime chrome mask (Shenzhen Qingyi Photomask Ltd., Singapore, Singapore) using direct laser writer µPG 101 (Heidelberg Instruments Mikrotechnik GmbH, Heidelberg, Germany). At first, the negative photoresist AZnLOF 2020 (Microchemicals GmbH, Ulm, Germany) was coated on 4-inch borofloat glass wafer (Bonda Technology Pty Ltd., Singapore, Singapore) for 30 s at 2000 rpm following a soft bake (2 min, 110 °C). The wafer was UV-exposed at 200 mJ cm^−2^ with the above patterned mask using a mask aligner (EVG 620, EV Group, St Florian am Inn, Austria). After the post-exposure bake (1 min, 110 °C), the wafers were developed for 45 s in AZ726 MIF Developer (Microchemicals GmbH, Ulm, Germany) and were cleaned using a PlasmaPro 80 (Oxford Instruments, Bristol, UK) to remove photoresist residues. A thin layer of Ti (10 nm) and Au (200 nm) was deposited on to the wafers with a Temescal FC-2000 electron beam evaporator (Ferrotec, Santa Clara, CA, USA). Lift-off was then performed overnight in Remover PG (Microchemicals GmbH, Ulm, Germany) at room temperature to reveal the gold coated circular microelectrode structures. In the second step, a well structure composed of polydimethylsiloxane (PDMS) was prepared by punching holes of 6 mm diameter into the cured PDMS. The PDMS was prepared by curing activated silicon elastomer solution (Sylgard^®^ 184, Dow Midland, MI, USA) for 1 h at 65 °C. The cured PDMS with wells was aligned manually to the circular microelectrode structures and bonded thermally at 65 °C for 2 h.

The device was then functionalized by biotin–avidin chemistry (Figure S1). First, 20 µL of 250 µg mL^−1^ biotin-BSA (Thermo Fisher Scientific, Seventeen Mile Rocks, Australia) were adsorbed directly on the electrodes for 2 h followed by 20 µL of 100 µg/mL streptavidin (Thermo Fisher Scientific, Seventeen Mile Rocks, Australia) and 20 µL of 100 µg/mL biotinylated-Jacalin (Vector Laboratories, Burlingame, CA, USA) were sequentially added to each well and incubated for 1 h at room temperature. To prevent drying out, the electrodes were sealed during the incubation steps. The electrodes were thoroughly washed with 1× PBS in-between each incubation step to remove any unbound biomolecules. Prior to the antigen incubation, 1% BSA in PBS was added to each well for 1 h as a blocking step.

### 2.9. SERS Nanotags Synthesis

The core sodium citrate-coated gold nanoparticles (AuNPs) were synthesized according to the citrate synthesis by Frens [29] involving citrate reduction of gold (III) chloride trihydrate (HAuCl_4_, Sigma-Aldrich Pty Ltd., North Ryde, Australia ). Initially, 100 mL of HauCl4 solution were boiled in water (0.01% *w/v*) with the addition of 1 mL of 1% trisodium citrate dehydrate (Univar Solutions, Downers Grove, IL, USA) and under constant stirring for 20 min. The synthesized AuNP were spherical with a diameter of ~60 nm diameter (Figure S2, [30]). Subsequently, 10 µL of 1 mM DTNB (5,5′-dithiobis (2-nitrobenzoic acid)) and 2 µL of 1 mM N-hydroxysuccinimide ester (DSP, Sigma-Aldrich Pty Ltd., North Ryde, Australia) were added to 1 mL of synthesized AuNPs and incubated at room temperature for 5 h with gentle shaking. After the incubation, the mixture was centrifuged at 5400× *g* for 10 min and resuspended in 20 µL of 0.1 mM phosphate-buffered saline (PBS, pH 7.4) buffer. The PBS buffer containing the AuNPs conjugated Raman reporters was mixed with 500 µg of anti α-C9 antibody and incubated at room temperature for 30 min following a centrifugation at 600× *g* for 6 min to remove unbound antibody. The SERS nanotags were then re-suspended in 0.1% bovine serum albumin (BSA, Sigma Aldrich, USA).

### 2.10. C9 Sample Preparation for EndoScreen Chip Assay

Patient samples and C9 protein purified from human serum were denatured prior to use. The denaturation buffer was 40 mM of Tris buffer (Thermo Fisher Scientific, USA) with 2% of sodium dodecyl sulphate (Sigma-Aldrich Pty Ltd., North Ryde, Australia, USA), 10% of Triton buffer and 40 mM of dithiothreitol (Sigma-Aldrich Pty Ltd., North Ryde, Australia) in ultra-pure water. The denaturation buffer was added to the sample at volume ratio of 1:1 and incubated at 65 °C for 30 min. After denaturation, the mixture was alkylated by the addition of 1 M of iodoacetamide (Bio-Rad Laboratories, Gladesville, Australia) to make up a final concentration of approximately 100 mM and incubated for 30 min at ambient temperature and protected from light. The denatured patient samples were diluted 100 times in 1× PBS (pH 7.2) and stored at −20 °C.

### 2.11. EndoScreen Chip Assay

Each prepared diluted patient sample was split into three equal volumes (50 µL) and added to three separate wells of the microfluidic device. The device was then connected to a signal generator 333510B (Agilent Technologies, Mulgrave, Australia). Incubation of the sample was carried out under an applied alternating current electrohydrodynamics (ac-EHD) nanomixing (f = 500 Hz, Vpp = 800 mV, t = 30 min) protocol that we have previously developed to improve detection specificity and sensitivity [28]. Next, the wells were washed with wash buffer (0.1% BSA, 0.01% Tween-20 in PBS) and incubated with 20 µL of SERS nanotags under the same ac-EHD conditions as above, but for 20 min. The wells were washed again with the wash buffer and the microfluidic device was stored overnight at 4 °C prior to the SERS mapping. Sample handling and washing steps were performed manually using a pipette.

SERS mapping of the microfluidic device was performed by a WITec Alpha 300 R confocal Raman microscope with a HeNe laser (32 mW, 633 nm) with a 20× microscopic objective. The acquisition parameters were 0.1 s integration time, step size of 1 µm and a mapping area of 60 µm × 60 µm (60 pixels × 60 pixels). Initial calibration for the instrument was performed by measuring the Raman intensity of the silicon substrate that produces a first~-order photon peak at ~520 cm^−1^. The raw Raman spectral data containing fluorescence and background noise were processed using a fifth-order polynomial fitting method developed by the Zhao and co-workers [31]. Each experiment was performed in triplicates and the intensity graph corresponding to the concentration of the C9 protein was plotted by taking the average of the peak Raman shift intensity of DTNB (1335 cm^−1^).

### 2.12. Statistical Analysis

Cohort analyses were conducted in GraphPad Prism 8 and IBM SPSS Statistics 23. To adjust for normality, JAC-C9 Raman intensity was transformed using a natural log, then converted to z-scores. BMI and heartburn/reflux history were entered as ordinal data into multinomial logistic regression based on increasing severity with population demographics [32]. Receiver operating characteristics (ROC) curves and confidence intervals were generated in JMP PRO (15.2.1, SAS Institute, Cary, NC, USA). For the positive predictive value, conversion from percentage points to percentages were calculated by the formula (percentage change/starting percentage) × 100%.

## 3. Results

### 3.1. Validation of New C9 Monoclonal Antibody

**Figure 1:**
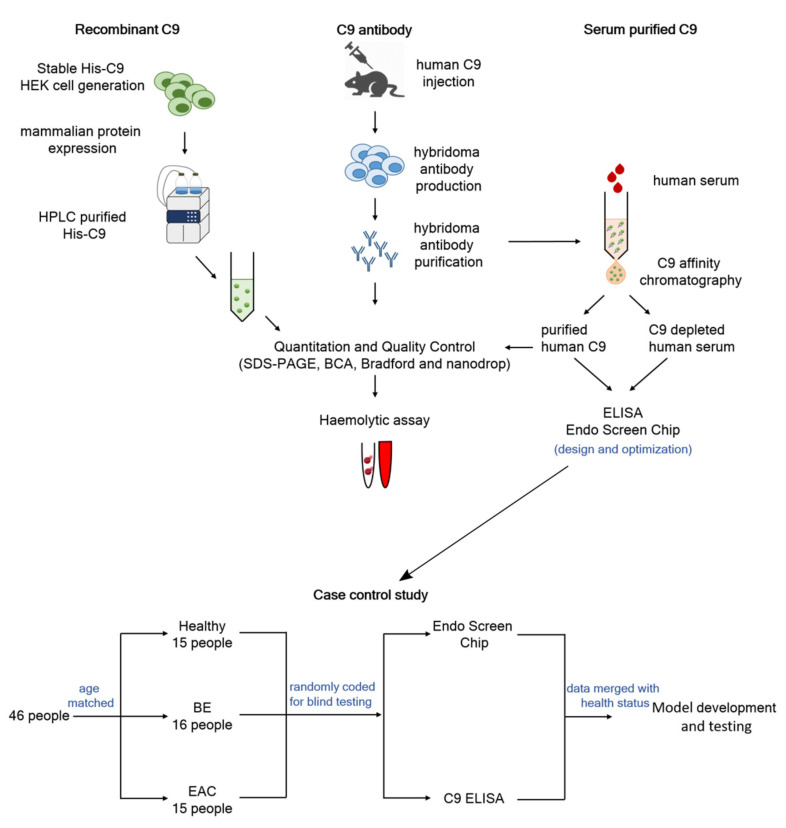
Study overview. High quality reagents including recombinant C9, C9 antibody and serum purified C9 were generated and used to develop C9 ELISA and EndoScreen Chip. The newly established assays were evaluated in a case-control cohort. Logistic regression was used to develop diagnostic algorithms for predicting BE or EAC, by combining blood markers with risk factors.

Immunoassays are highly reliable and cost-effective clinical tools used for the high throughput analysis of serum biomarkers; however, an ongoing supplying of high quality reagents is essential. In order to develop a lectin immunoassay for a glycoform of C9 that binds to Jacalin lectin (JAC-C9), we developed renewable, quality reagents for the assays (Figure 1), including a new monoclonal anti-human-C9 antibody, m26 3C9 (Figure S1A,B) and a recombinant C9 protein expressed in a mammalian cell system (Figure S1C,D). The antibody was verified to detect human recombinant and serum purified C9, with high affinity towards native C9 and a lower affinity towards denatured C9 (Figure S1E). In addition, we generated C9-depleted serum for use as background matrix in the immunoassay. Successful depletion was verified using a red blood cell lysis assay; depleted serum lowered lysis to near background, while add-back of C9 elevated lysis to level of normal human serum (Figure S1F).

**Figure 2:**
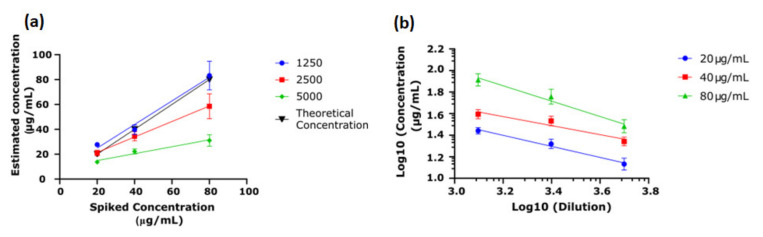
Validation of C9 direct ELISA. His-C9 was expressed and purified for use as the standard, and serum purified C9 was spiked into C9-depleted serum at three concentrations (20 µg/mL, 40 µg/mL and 80 µg/mL). (**a**) Concentration estimated by the ELISA was plotted against the spiked concentration. Dilutions of 1/1250 predicted the theoretical concentration accurately while higher dilutions underestimated the theoretical concentration when samples were spiked at 40 µg/mL and 80 µg/mL (*n* = 3). (**b**) Parallelism was determined by log10 calculation of dilution vs. estimated concentration of the spiked sample, demonstrating proportionate estimates of concentration at differing dilutions (*n* = 3).

To inform on starting parameters for JAC-C9 lectin immunoassay, we first evaluate the performance of m26 3C9 in a direct ELISA. The accuracy, repeatability, linearity and range were calculated according to the US Food and Drug Administration Q2 (R1) Validation Guidelines [33], using recombinant His-C9 in the standard curve. Firstly, the optimal serum dilution for measurement accuracy was determined using purified C9 spiked into C9-depleted serum at three concentrations (20 µg/mL, 40 µg/mL and 80 µg/mL). As shown in Figure 2a, at a serum dilution of 1/1250, the ELISA accurately measured the concentration of C9 in the spiked sample; however, at higher dilutions, the ELISA underestimated the concentration of the spiked serum. Therefore, serum dilution was fixed at 1/1250. Repeatability was calculated following recommendations by Andreasson and colleagues [34]; the co-efficient of variation (CV) was 3.8%, 5.6% and 4.3% for 20 µg/mL, 40 µg/mL and 80 µg/mL, respectively (*n* = 5). Linearity was used to demonstrate that the result obtained was directly proportional to the concentration. Given the accuracy of the 1/1250 dilution, it was clear that the result obtained was directly proportional to the concentration (Figure 2a); however, linearity was also assessed for the remaining concentrations (Figure 2b) [34]. Despite the noted underestimate of concentration at high serum dilutions, the concentration determined was still proportional to the actual concentration. Furthermore, since the His-C9 standard was used in combination with spiked serum purified C9, these experiments demonstrate that recombinant His-C9 is a suitable alternative to serum purified C9 for use as an assay standard.

### 3.2. Establishing the Endo Screen Chip

**Figure 3:**
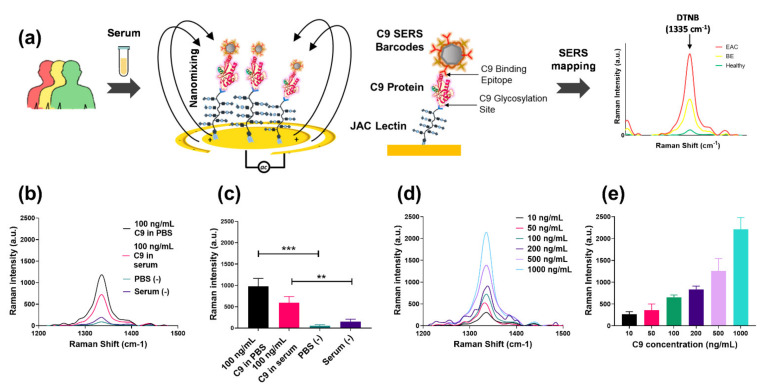
Establishing the EndoScreen Chip. (**a**) Schematic workflow of the chip assay for JAC-C9 detection. JAC-mediated glycoprotein isolation from denatured serum samples and C9 labelling by SERS barcode-tagged anti-C9 antibody is performed under the stimulation of a nanoscopic fluid flow. JAC-C9 is detected by SERS mapping, where the Raman reporter DTNB that is conjugated to the SERS barcodes provides a characteristic Raman peak at 1335 cm^−1^. (**b**) Raman spectra of 100 ng/mL C9 in PBS (black), 100 ng/mL C9 in diluted serum (pink), blank PBS (cyan) and diluted serum (purple). (**c**) Corresponding averaged Raman signal intensity of DTNB (1335 cm^−1^). (**d**) Raman spectra and (**e**) averaged Raman signal intensity of DTNB (1335 cm^−1^) obtained for designated C9 concentrations spiked in diluted serum. The error bars are the standard error of three replicates. ** *p* < 0.01 and *** *p* < 0.001.

Attempts at microplate-based JAC-C9 ELISAs were unsuccessful, possibly due to high background and low signal-to-noise ratio, which had been previously observed [35]. Therefore, we developed the EndoScreen Chip (Figure 3a), which integrates highly sensitive nanoparticle barcodes, alternating current electrohydrodynamic (ac-EHD)-induced nanofluidic mixing, surface-enhanced Raman spectroscopy (SERS) and an array of 28 wells supporting parallel sample analysis (Figure S3), with each technical replicate analyzed in a separate well. The ac-EHD-induced nanofluidic mixing previously developed by our group [20] acts in nanometer proximity to the JAC-functionalized electrode to (i) stimulate collisions of JAC-C9 with the sensor surface, (ii) accelerate the binding of the SERS barcodes with the captured JAC-C9 and (iii) reduce sensor fouling through the induced shear forces that remove non-specifically adsorbed non-target molecules. Furthermore, we chose SERS as the detection mode due to its high sensitivity, high photo stability of the SERS reporters and narrow spectral width of the Raman reporter peaks [36].

As depicted in Figure 3a, the asymmetric gold electrodes in each well were functionalized with JAC to capture glycoproteins that bind JAC lectin, including JAC-C9. Subsequent incubation with anti-C9 antibody conjugated SERS nanoparticle barcodes specifically labels the captured JAC-C9 molecules by the C9 protein epitope. The SERS barcodes were also modified with the Raman reporter 5,5′-dithiobis 2-nitrobenzoic acid (DTNB). Upon laser excitation at 633 nm, DTNB provided a characteristic Raman shift at 1335 cm^−1^, and its intensity was used for quantification of JAC-C9.

The specificity of EndoScreen Chip was investigated using solutions of 100 ng/mL purified C9 in PBS or in diluted C9-depleted serum (Figure 3b). A strong Raman signal at 1335 cm^−1^ was observed for C9-containing samples, significantly higher than the negligible signals detected in the respective buffer or serum negative controls (*p* < 0.01, *t*-test, Figure 3b,c). Promisingly, the presence of diluted serum matrix caused only a minor suppression on the Raman signal compared to PBS buffer, suggesting the applicability of the assay for clinical sample analysis. Next, we investigated the sensitivity of our assay by using serial dilutions of purified C9 in diluted C9-depleted serum (Figure 3d,e). The graphed characteristic Raman peak signals (1335 cm^−1^) in Figure 3e showed a strong correlation with JAC-C9 concentration (Pearson R^2^ = 0.9835, *p* < 0.0001). Based on a signal-to-noise ratio of 3, the JAC-C9 detection sensitivity was calculated to be 6.3 ng/mL of spiked purified C9.

### 3.3. Total C9 and JAC-C9 Are Elevated in EAC Patient Serum

To evaluate the performance of the EndoScreen Chip, we analyzed a subset of 46 serum samples from the Study of Digestive Health (SDH) [22]. We previously reported elevated serum JAC-C9 in EAC sera in this cohort using multiple reaction monitoring (MRM) mass spectrometry [15], but the total serum C9 level had not yet been analyzed. The patients in the SDH study were diagnosed by endoscopy pathology as Healthy, BE or EAC. The selected cohort was balanced for age across the three groups, but differed in heartburn/reflux history, while body mass index (BMI) showed a trend to be higher in EAC (Table 1).

**Figure 4:**
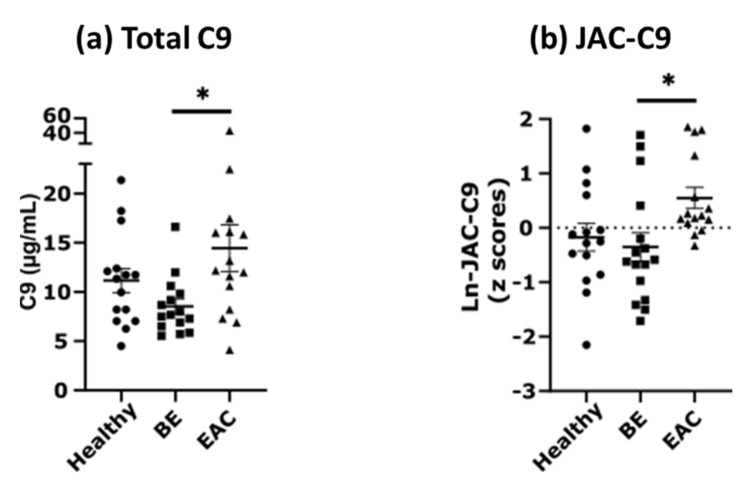
Serum C9 and JAC-C9 are increased in EAC in a cohort of 46 samples. (**a**) Serum C9 concentration was determined using direct ELISA with m26 3C9 antibody. Patients diagnosed with EAC show significantly increased C9 concentration relative to BE (one-way ANOVA with Tukey’s multiple comparisons, *p* = 0.0323). (**b**) JAC-C9 was determined using EndoScreen Chip. Patients diagnosed with EAC show significantly increased JAC-C9 relative to BE (one-way ANOVA with Tukey’s multiple comparisons, *p* = 0.0279). *, *p* < 0.05.

EndoScreen Chip analysis showed that serum JAC-C9 is significantly elevated in EAC compared to BE (one-way ANOVA with Tukey’s multiple comparisons, *p* = 0.0279), which is consistent with our lectin pulldown coupled mass spectrometry results in this cohort [15] and thereby validates the new EndoScreen Chip. Interestingly, total serum C9 quantified by the new monoclonal m26 3C9 ELISA was also significantly elevated in EAC compared to BE (one-way ANOVA with Tukey’s multiple comparisons, *p =* 0.0323, Figure 4a). Although levels of both C9 and JAC-C9 were also increased in EAC compared to Healthy, the difference was not statistically significant in this limited cohort. These results confirm the validity of the EndoScreen Chip for JAC-C9 measurement, and provided the first data suggesting elevated serum C9 in EAC.

### 3.4. Serum C9 Biomarker Panel Improves the Detection of EAC over Clinical Risk Factors Alone

While ultimately the EndoScreen Chip is envisaged to measure a panel of biomarkers in a multiplex signature, as proof-of-concept, we made use of the current cohort data to determine if the current small panel of C9 and JAC-C9 could assist in patient stratification in the primary care setting. Specifically, we asked if the blood markers help the primary care physician identify which patients are more likely to have BE or EAC and should therefore be prioritized for endoscopic investigation, given the presenting clinical risk factor data.

**Figure 5:**
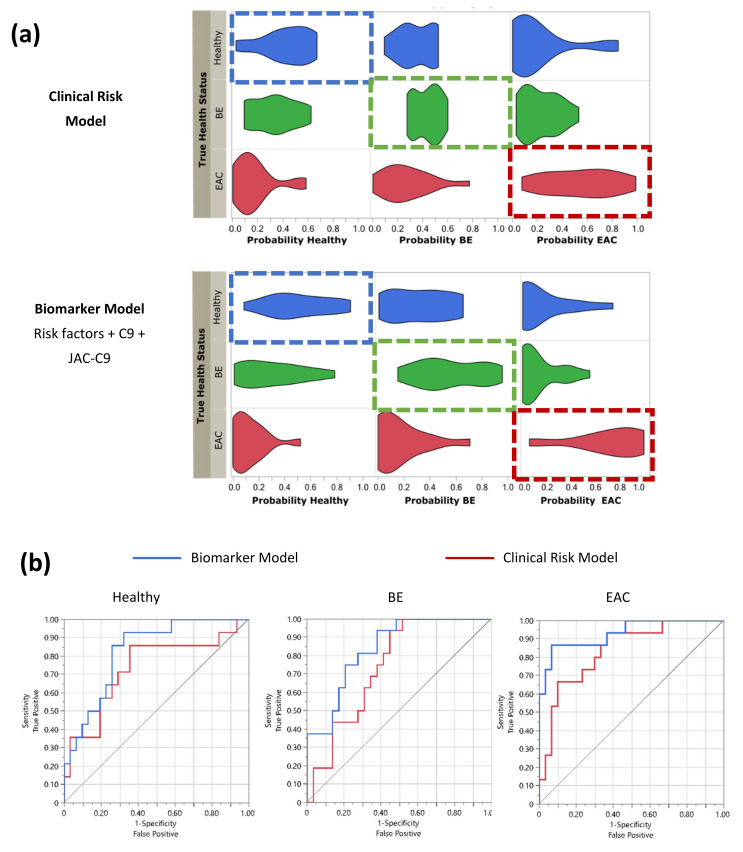
Serum biomarkers improve patient stratification in the study cohort. Logistic regression modelling was conducted on the cohort (*n* = 46) for classification as Healthy, BE or EAC, using patient risk factors of BMI, age and heartburn/reflux history alone (Clinical Risk Model), or risk factors plus serum biomarkers C9 and JAC-C9 (Biomarker Model). (**a**) Probability of health status was plotted as a violin plot against true classification. Boxes indicate the true health status, shift to the right indicates improved classification. (**b**) Receiver operating curve (ROC) for correctly predicting health status for Clinical Risk Model (red) vs. Biomarker Model (blue). See Table 2 for statistics.

Multinomial logistic regression was conducted on the presenting clinical data and the biomarker data to predict the dependent variables (BE or EAC diagnosis) relative to Healthy. The baseline probability was established by inputting the presenting clinical risk parameters of age, BMI and heartburn/reflux history (Table 1) together as a forced forward entry model (Clinical Risk Model). A second model was then developed by incorporating the blood marker panel data for C9 and JAC-C9 into the baseline model (Biomarker Model, Figure 5). Probabilities of correct prediction of disease status from the multinomial logistic regression analysis were visualized as violin plots (Figure 5a). The right shift in the probability of true classification in the Biomarker Model compared to the Clinical Risk Model showed that the addition of blood biomarkers improved the stratification of patients into correct diagnoses (Figure 5a). The diagnostic performance of the two models was examined by receiver-operating curve (ROC) analysis for correctly classifying Healthy, BE and EAC from the cohort (Figure 5b, Table 2). For all three diagnostic categories, the Biomarker Model increased the area under curve (AUC) relative to Clinical Risk Model; however, only EAC detection reached statistical significance when comparing the AUC between the two models (Table 2). Furthermore, the additions of C9 and JAC-C9 biomarkers are statistically significant (*p* = 0.0170 and *p* = 0.0073, respectively, *p* = 0.0012 as a group, Likelihood Ratio Test, Table S1).

**Figure 6:**
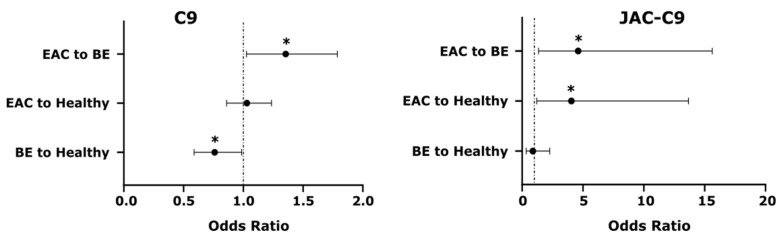
Serum C9 and JAC-C9 show different odds ratios for differentiating between pairs of health conditions in the Biomarker Model. * Wald’s test, *p* < 0.05.

While the above models included both JAC-C9 and C9, we were interested to know if the two markers independently contribute to the predictive model. As JAC-C9 is a glycoform (subset) of total C9, it was possible that the two measures provided similar information. Interestingly, a paired samples correlation between C9 and JAC-C9 showed that these two variables are not significantly correlated in this dataset (*r* = 0.119, *p* = 0.432, *n* = 46). Therefore, we further investigated the contribution of individual markers using odds ratios and Wald statistic for pairs of health conditions (Figure 6). Both C9 and JAC-C9 contributed significantly to the distinction between EAC and BE, but JAC-C9 had a much higher odds ratio (OR = 4.6; 95% CI: 1.6–15.6; Wald statistic, *p* = 0.014) compared to total C9 (OR = 1.4; 95% CI: 1.0–1.8; Wald statistic, *p* = 0.032). Furthermore, JAC-C9, but not total C9, contributed to the prediction of EAC relative to Healthy (OR = 4.1; 95% CI: 1.2–13.7; Wald statistic, *p* = 0.024). On the other hand, total C9, but not JAC-C9, contributed to the prediction of BE relative to Healthy (Wald statistic, *p* = 0.039), albeit with a modest odds ratio (OR = 0.8; 95% CI: 0.6–1.0). The different diagnostic properties of JAC-C9 and total C9 validate our initial glycoproteomics approach and the development of a glycoform specific JAC-C9 assay.

## 4. Discussion

Despite the development of clinical risk prediction models and endoscopic surveillance protocols, the ability to diagnose EAC earlier has not appreciably improved over the past few decades [4]. On the other hand, the demand for endoscopies is escalating due to population ageing and poor lifestyle factors which lead to gastrointestinal pathologies. Endoscopy is an invasive procedure requiring expert clinicians and significant healthcare resources, but health economics evaluations indicate a lack of precision in patient selection. For example, a recent Dutch study concluded the overuse of endoscopy surveillance for BE and BE with low grade dysplasia is associated with substantial costs to the Dutch health system ($53 million per year) for very marginal and uncertain benefits [37]; a 2018 Australian government report indicates 14% of upper endoscopies in adults offered little benefit [38]. With future incorporation of additional validated EAC biomarkers [16] to a panel test, the EndoScreen Chip technology reported in this study has the potential to improve patient triage for endoscopies when used as a first-line blood test for screening and surveillance of patients with risk factors of BE and EAC. For point-of-care application, a compact device that uses an open source electrical board and smartphone-integrated fluorescence detection could be adopted [39].

A blood biomarker panel test has several advantages that directly address key determinants of the cost-effectiveness of EAC screening and BE surveillance [40]. A simple, low-cost blood test conducted by the primary care physician will promote greater testing, which may help triage patients at higher risk and improve the diagnostic yield of those who proceed to endoscopy. Additionally, use of an inexpensive liquid biopsy will enable more frequent testing, potentially leading to detection at an earlier stage. From the patient and compliance viewpoint, blood testing is familiar and commonly accepted. Indeed, a recent survey of 554 Dutch population respondents returned a strong preference of non-invasive (blood or breath) tests over endoscopic or capsule-based tests, provided the test is sufficiently sensitive [41]. Furthermore, a single blood draw may be used for multiple blood tests in the pathology laboratory. As older age increases the risk of multiple cancers, a routine blood draw for pan-cancer risk stratification is an attractive option, if a sensitive test exists for pan-cancer screening. This possibility was recently demonstrated by a large, multi-center trial with >9900 participants baseline tested using the multi-analyte CancerSEEK blood test, with positive cases followed by positron emission tomography–computed tomography (PET-CT) for diagnosis and localization of the cancer, if present [42]. In our scenario, detection of high risk patients using EndoScreen Chip will be followed with endoscopic diagnosis and treatment if required.

The current proof-of-concept development of EndoScreen Chip used complement component 9 (C9) as an example EAC serum biomarker. C9 is a circulating glycoprotein with complex glycosylation patterns [43]. In addition to our work in EAC, proteomic and glycoproteomic cancer biomarker discovery studies have reported plasma/serum C9 as a biomarker for colorectal cancer, gastric cancer, squamous cell lung cancer and glioblastoma [44,45,46,47]. While we have confirmed elevated C9 glycoforms in multiple EAC cohorts using lectin-pulldown-coupled quantitative mass spectrometry [15,16], this is the first report of elevated total serum C9 in EAC. While additional EAC cohort evaluation for total serum C9 remains to be conducted, the multiple independent reports of elevated serum C9 or glycoforms of C9 in multiple cancers support the mounting evidence for complement system dysregulation in cancer [48]. Future evaluation and comparison of the C9 changes in different cancers will be required to determine if JAC-C9 and the final biomarker panel for EndoScreen Chip are EAC-specific or overlap with another cancer type.

In the present cohort, we found that JAC-C9 has a much higher odds ratio for EAC compared to total C9, which affirms the sub-proteome approach to biomarker discovery. We focused on glycoproteins and chose lectin as an affinity agent, because the results could be readily translated to lectin immunoassays. To overcome the high background, and low sensitivity observed with a conventional lectin ELISA, we devised a microfluidic chip strategy—the EndoScreen Chip. An electric field induced nanoscopic fluid flow in proximity to the surface-immobilized JAC was employed to remove weakly bound molecules, while signal enhancement by localized surface plasmon resonance allows high sensitivity detection. Working synergistically, the nanofluidic mixing and SERS read-out were instrumental to the achieved limit of detection of 6.3 ng/mL in patient serum. Following this proof of concept work, the EndoScreen Chip will be expanded to multiplex a panel of glycoprotein biomarkers. This can be achieved either by using additional antibodies with different SERS tags to detect other JAC-binding protein biomarkers such as JAC-GSN and JAC-PON3 [16], or by adding other lectins to the other channels, for detection with C9, or other antibodies. Each of the new biomarker lectin immunoassays will need to be separately validated prior to multiplexing, ensuring lack of cross-reactivity. The final multiplex EndoScreen Chip will then be evaluated on a larger cohort.

Although the current technology study is limited to proof-of-concept of JAC-C9 measurement and data modeling in a single cohort, previous health economic evaluations in large cohorts in real-world settings confirmed the value of biomarkers in risk-stratifying patients for BE screening [49,50]. After development of the multiplex EndoScreen Chip, future modelling will use simulation techniques to assess the benefits, harms and costs of screening with this new technology, taking into account the limitations of direct analysis of small samples and the uncertainty arising.

## 5. Conclusions

In conclusion, the sensitive microfluidic pre-endoscopy blood biomarker EndoScreen Chip test has the potential to transform BE management and EAC detection. Although the development of a multiplexed panel and clinical trials are still needed, ultimately, the ability to stratify patients for EAC risk based on blood markers and clinical risk factors will increase patient compliance in screening programs and allow the most effective utilization of endoscopy resources.

## Figures and Tables

**Table 1:** Characteristics of the selected cohort.

Parameter	Category	Healthy	BE	EAC	*p* Value (Healthy vs. BE vs. EAC)
Number		15	16	15	
Age (years)	Median	64.39	61.89	61.72	0.146 ^a^
	Range	56–75	52–75	53–74	
BMI	Healthy wt (<25)	10.9% (5)	6.5% (3)	2.2% (1)	0.054 ^b^
	Overweight (<30)	10.9% (5)	23.9% (11)	10.9% (5)	
	Obese I (<35)	10.9% (5)	4.3% (2)	17.4% (8)	
	Obese II (<40)	0.0% (0)	0.0% (0)	0.0% (0)	
	Obese III (≥40)	0.0% (0)	0.0% (0)	2.2% (1)	
Heartburn and Reflux History	Never	11.1% (5)	4.4% (2)	2.2% (1)	0.005 ^b^
	<Once/month	6.7% (3)	6.7% (3)	2.2% (1)	
	Monthly (few times/month)	8.9% (4)	13.3% (6)	2.2% (1)	
	Weekly (few times/wk)	2.2% (1)	0.0% (0)	15.6% (7)	
	Daily	2.2% (1)	11.1% (5)	11.1% (5)	

Percentage calculated is percentage relative to all cases. Brackets are the number of counts of each class. ^a^ Kruskal–Wallis Test. ^b^ Fisher’s Exact Test.

**Table 2:** Comparison of health status classification by each model.

Predictive model	Health Status: Healthy				
Predictor	AUC	StdError	Lower 95%	Upper 95%	*Prob > ChiSq*
Clinical Risk Model	0.7350	0.0886	0.5322	0.8712	
Biomarker Model	0.8272	0.0621	0.6714	0.9181	
*Difference*	*−0.092*	*0.0635*	*−0.217*	*0.0323*	*0.1465*
	**Health Status: BE**				
Predictor	AUC	StdError	Lower 95%	Upper 95%	*Prob > ChiSq*
Clinical Risk Model	0.7435	0.0726	0.5788	0.8595	
Biomarker Model	0.8405	0.0579	0.6934	0.9247	
*Difference*	*−0.097*	*0.0690*	*−0.232*	*0.0382*	*0.1598*
	**Health Status: EAC**				
Predictor	AUC	StdError	Lower 95%	Upper 95%	*Prob > ChiSq*
Clinical Risk Model	0.8378	0.0624	0.6774	0.9270	
Biomarker Model	0.9311	0.0411	0.7936	0.9794	
*Difference*	*−0.093*	*0.0432*	*−0.178*	*−0.009*	0.0309 *

AUC, area under the curve; * *p* < 0.05.

## Data Availability

The data presented in this study are available in this article and the supplementary materials.

## Supplementary Materials

The following are available online at https://www.mdpi.com/article/10.3390/cancers13122865/s1. Figure S1: Production and purification of C9 and antibody. Figure S2: Scanning electron microscopy image of synthesized AuNP of ~60 nm diameter. Figure S3: Endo Screen Chip design and functionalization. Table S1: Independent contribution of each predictor in Biomarker Model by Effect Likelihood Ratio Test.
