# Supplementary material for: Development of EndoScreen Chip, a Microfluidic Pre-Endoscopy Triage Test for Esophageal Adenocarcinoma
Source: Cancers (Basel). 2021 Jun 8;13(12):2865. doi: 10.3390/cancers13122865 (PMC8229863; doi:10.3390/cancers13122865)
Supplement: Supplementary file 1 [file cancers-13-02865-s001.zip › cancers-1227683-supplementary.pdf]

Supplementary Information for

Development of EndoScreen Chip, a microfluidic pre-endoscopy triage test for esophageal adenocarcinoma

Julie A. Webster[1], Alain Wuethrich[2], Karthik B. Shanmugasundaram[2], Renee S. Richards[1], Wioleta M. Zelek[3], Alok K. Shah[1], Louisa Gordon[1], Bradley J. Kendall[1,4], Gunter Hartel[1], B. Paul Morgan[3], Matt Trau[2,5] and Michelle. M. Hill[1,4*]

Figure S1: Production and purification of C9 and antibody.

Figure S2: Scanning electron microscopy image of synthesized AuNP of ~60 nm diameter

Figure S3: Endo Screen Chip design and functionalization.

Table S1: Independent contribution of each predictor in Biomarker Model by Effect Likelihood Ratio Test

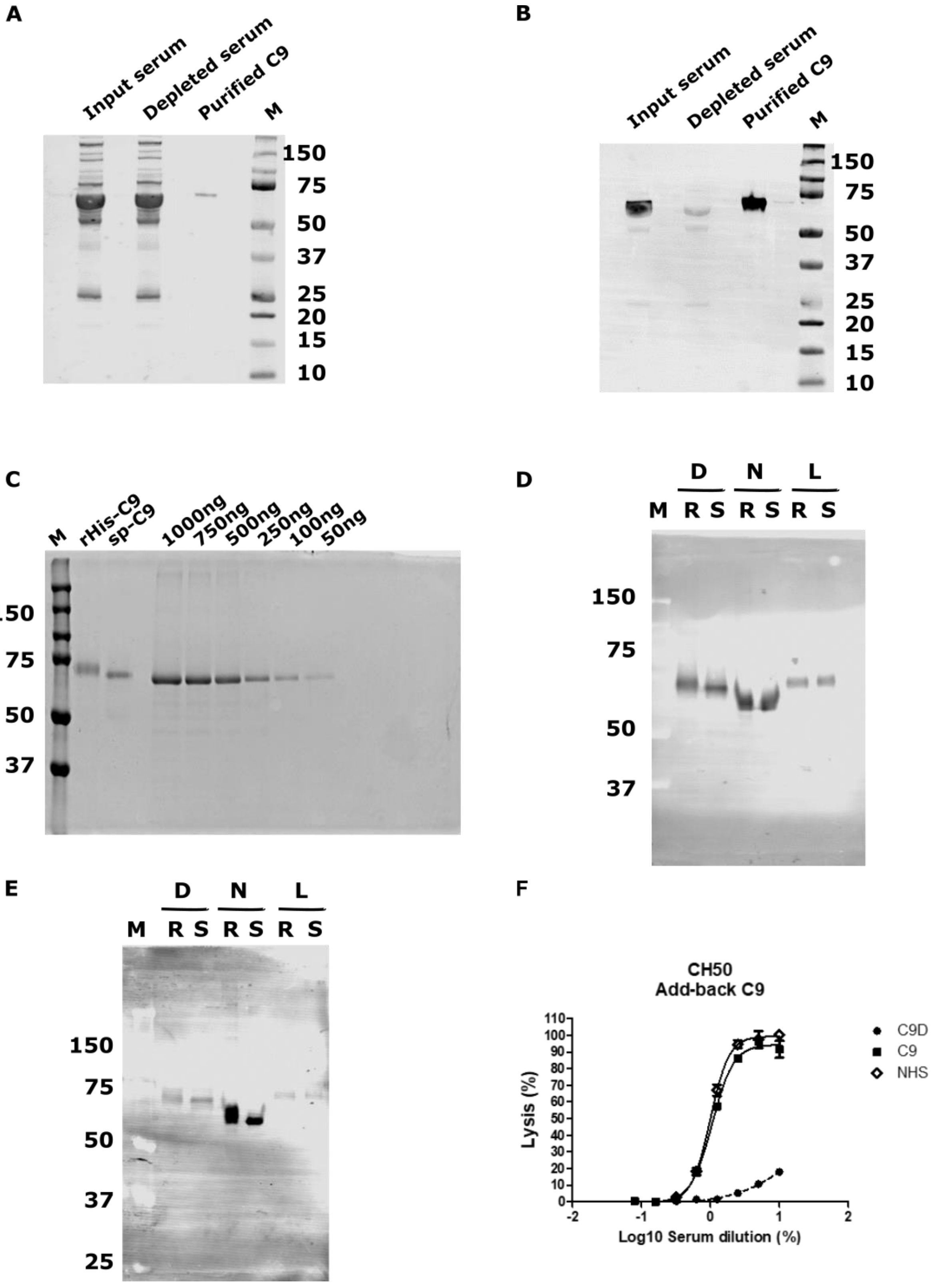

S

Figure S1. Production and purification of C9 and antibody. *(A) Human serum was depleted of C9 using mAb26. Purity was checked using SDS-Page. Only one band was detected after purification and staining of the membrane using REVERT total protein stain, confirming no contaminants were present. (B) Western blot using mouse anti-C9 antibody (Abcam) confirmed the protein purified was C9 with minimal C9 detected in the depleted serum. (C) Recombinant C9 was produced in HEK cells*

then purified using FPLC on an AKTA system. Purified recombinant His-C9 and human C9 resolved on 10% PAGE gels under reducing conditions, stained with Colloidal Coomassie. Concentration was quantitated relative to dilutions of BSA. M: protein molecular weight marker. Gel shows that both types of C9 had no contaminating C9 or degradation. (D) 125 ng of C9 was resolved on and SDS-Page then analyzed by western blot. Recombinant C9 (R) was confirmed using mouse anti-C9 (Abcam) and was compared to serum (S) purified C9. Protein was either denatured (D=reduce/heat) or natured (N=not reduced/no heat) or denatured as per LeMBA (L) buffer. (E) mAb 26 antibody was characterized for detection of various preparations of C9 as per (D). mAb 26 had a high affinity for natured C9 relative to denatured C9, hence was highly suited for immunoprecipitation from serum. (F) mAb 26-purified C9 was titrated back into 10% C9 depleted serum to demonstrate the restored haemolysis. The dashed lines correspond to the C9 depleted serum (C9D) and open symbol to normal human serum (NHS). The assays were performed a minimum of three times with the comparable outcome. The error bars are standard errors of triplicates.

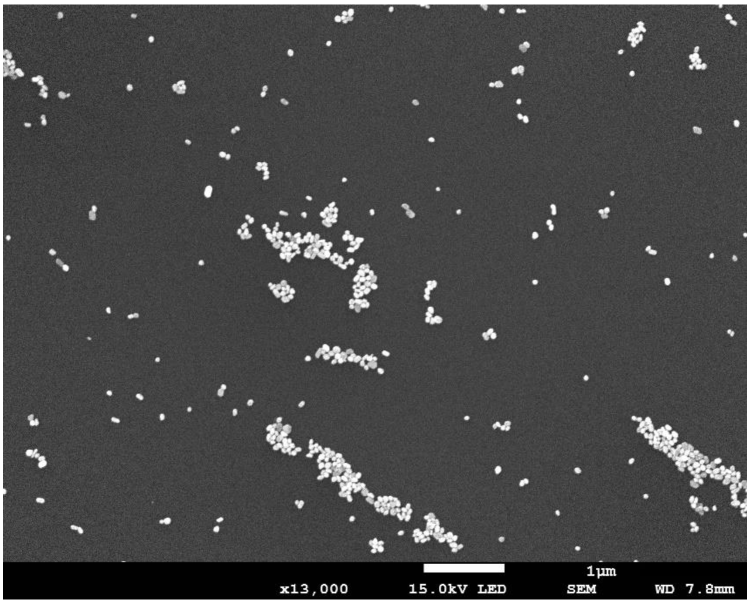

Figure S2. Scanning electron microscopy image of synthesised AuNP of ~60 nm diameter. *The scale bar is 1 μm.*

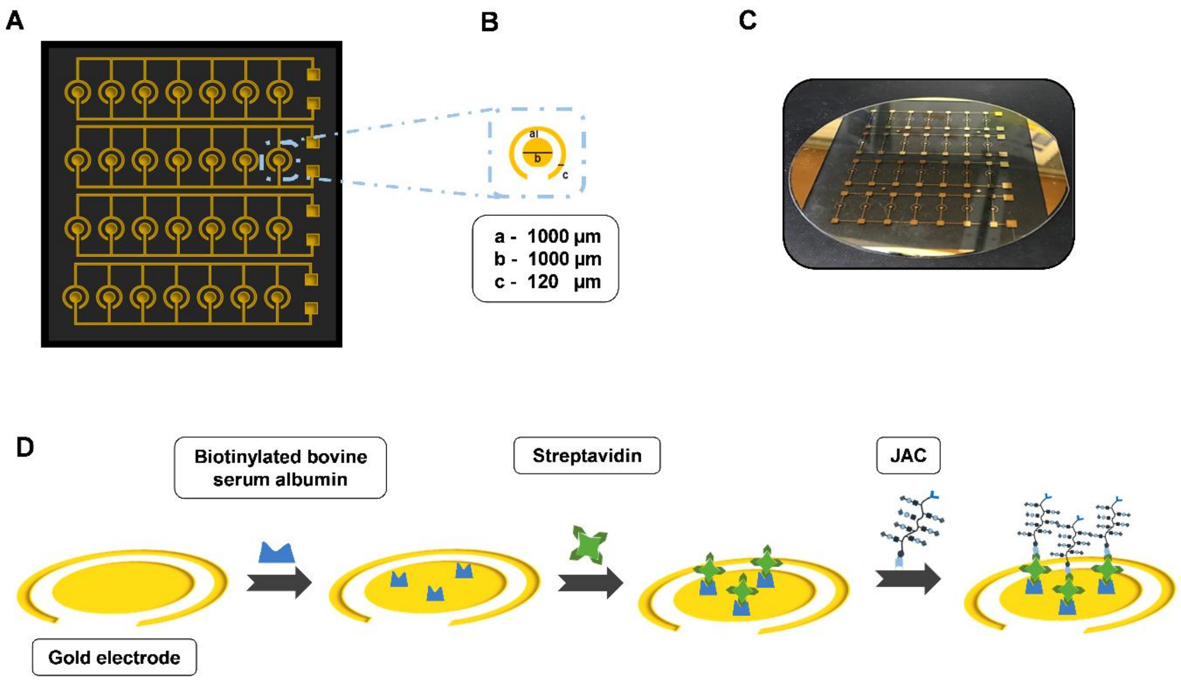

Figure S3. Endo Screen Chip design and functionalization. *(A) The Endo Screen Chip consists of an array of 28 electrode wells arranged in four rows to seven electrode wells each. (B) Each electrode was made of an inner circular electrode (1000 µm in diameter) and outer ring electrode (120 µm wide) that were separated by 1000 µm. The circular electrode and ring electrode acted as working and counter electrodes, respectively. (C) A photograph of the Endo Screen Chip. (D) Electrode functionalization with JAC using a biotin-streptavidin conjugation process.*

Table S1. Independent contribution of each predictor in Biomarker Model by Effect Likelihood Ratio Test

**Parameter**	**DF**	**L-R ChiSquare**	**Prob>ChiSq**
Age	2	3.48	0.1753
BMI	2	8.64	0.0133*
heartburn_reflux_history	2	11.87	0.0027*
C9	2	8.15	0.0170*
JAC_C9	2	9.84	0.0073*
C9 and JAC_C9	4	18.10	0.0012*

DF, Degree of Freedom

L-R ChiSquare, Likehlihood Ratio ChiSquare

* $p<0.05$ by ChiSquare test
